# Quasispecies Analysis of SARS-CoV-2 of 15 Different Lineages during the First Year of the Pandemic Prompts Scratching under the Surface of Consensus Genome Sequences

**DOI:** 10.3390/ijms232415658

**Published:** 2022-12-10

**Authors:** Wahiba Bader, Jeremy Delerce, Sarah Aherfi, Bernard La Scola, Philippe Colson

**Affiliations:** 1 IHU Méditerranée Infection, 19–21 Boulevard Jean Moulin, 13005 Marseille, France; 2 Microbes Evolution Phylogeny and Infections (MEPHI), Institut de Recherche pour le Développement (IRD), Aix-Marseille University, 27 Boulevard Jean Moulin, 13005 Marseille, France; 3 Assistance Publique-Hôpitaux de Marseille (AP-HM), 264 rue Saint-Pierre, 13005 Marseille, France

**Keywords:** SARS-CoV-2, quasispecies, variant, next-generation sequencing, Marseille

## Abstract

The tremendous majority of SARS-CoV-2 genomic data so far neglected intra-host genetic diversity. Here, we studied SARS-CoV-2 quasispecies based on data generated by next-generation sequencing (NGS) of complete genomes. SARS-CoV-2 raw NGS data had been generated for nasopharyngeal samples collected between March 2020 and February 2021 by the Illumina technology on a MiSeq instrument, without prior PCR amplification. To analyze viral quasispecies, we designed and implemented an in-house Excel file (“QuasiS”) that can characterize intra-sample nucleotide diversity along the genomes using data of the mapping of NGS reads. We compared intra-sample genetic diversity and global genetic diversity available from Nextstrain. Hierarchical clustering of all samples based on the intra-sample genetic diversity was performed and visualized with the Morpheus web application. NGS mapping data from 110 SARS-CoV-2-positive respiratory samples characterized by a mean depth of 169 NGS reads/nucleotide position and for which consensus genomes that had been obtained were classified into 15 viral lineages were analyzed. Mean intra-sample nucleotide diversity was 0.21 ± 0.65%, and 5357 positions (17.9%) exhibited significant (>4%) diversity, in ≥2 genomes for 1730 (5.8%) of them. ORF10, spike, and N genes had the highest number of positions exhibiting diversity (0.56%, 0.34%, and 0.24%, respectively). Nine hot spots of intra-sample diversity were identified in the SARS-CoV-2 NSP6, NSP12, ORF8, and N genes. Hierarchical clustering delineated a set of six genomes of different lineages characterized by 920 positions exhibiting intra-sample diversity. In addition, 118 nucleotide positions (0.4%) exhibited diversity at both intra- and inter-patient levels. Overall, the present study illustrates that the SARS-CoV-2 consensus genome sequences are only an incomplete and imperfect representation of the entire viral population infecting a patient, and that quasispecies analysis may allow deciphering more accurately the viral evolutionary pathways.

## 1. Introduction

Severe acute respiratory syndrome coronavirus 2 (SARS-CoV-2) emerged in December 2019 in Wuhan, China, and is the seventh coronavirus known to infect humans [1]. In early 2020, this virus quickly spread around the world and was declared a pandemic in mid-March 2020 [2]. As of 2 October 2022, it was estimated to have caused 617,879,854 cases and be involved in 6,546,448 deaths worldwide (https://coronavirus.jhu.edu/map.html (accessed on 3 October 2022)). SARS-CoV-2 is an enveloped single stranded positive RNA virus and belongs to the genus *Betacoronavirus*. Its genome is 29,903 nucleotide-long (based on the genome of the Wuhan-Hu-1 isolate, GenBank Accession NC_045512.2) and harbors 14 open reading frames (ORFs) that encode 31 structural, non-structural, or regulatory/accessories proteins. These proteins include, in the order of their genes from the 5′ region to the 3′ region of the genome [3,4]: two large polyproteins, ORF1a and ORF1b, that are proteolytically cleaved by a virus-encoded protease into 16 non-structural, enzymatic proteins (NSP1-16) involved in viral replication and that notably comprise the RNA-dependent RNA polymerase, an endonuclease, and a helicase; four structural proteins including the spike (S) protein that binds to the angiotensin-converting enzyme 2 (ACE2) receptor of the host cells, the envelope (E) protein, the membrane (M) protein, and the nucleocapsid (N) protein, all these proteins being common to all coronaviruses and considered as major targets for the development of antiviral drugs and vaccines [3,5,6]; and eleven regulatory/accessory proteins, ORF3a, ORF3b, ORF3c, ORF3d, ORF6, ORF7a, ORF7b, ORF8, ORF9b, ORF9c, and ORF10. The SARS-CoV-2 genome is flanked by two untranslated regions (UTR) (5′UTR and 3′UTR).

RNA viruses replicate using their low-fidelity RNA-dependent RNA polymerase, which generates mutations at a high rate, estimated to be 10^−3^–10^−5^ substitutions/nucleotide/replication cycle [7]. For SARS-CoV-2, the mutation rate was estimated to be between 1.0 and 5.0 × 10^−6^/nucleotide/cycle, corresponding to ≈1.12 × 10^−3^/nucleotide/year, therefore ≈33.5 substitutions/genome/year [8]; or to be 6.7 × 10^−4^ substitution/nucleotide/year (20.0 substitutions/genome/year) [9]. It was observed to be ≈30.4 substitutions/genome/year (https://nextstrain.org/ncov/gisaid/global/6m?l=clock (accessed on 30 September 2022)) [10] or 9.8 × 10^−4^ substitutions per site per year [11]. Coronaviruses, among RNA viruses, have the particularity to harbor an enzymatic protein (NSP14) with correction/repair functions due to its 3′-5′ exonuclease activity, which is likely related to the large size of their genomes that are the largest among RNA viruses. Mutations that occur in the SARS-CoV-2 genome are nucleotide substitutions, deletions, or insertions, and some are non-synonymous, generating codon changes and amino acid substitutions, deletions, or insertions [12,13,14]. Another evolutionary mechanism of coronaviruses are recombinations [15], which have been increasingly reported in SARS-CoV-2 [16,17]. Altogether nucleotide changes may facilitate adaptation to new hosts and environments and may impact on the efficacy of vaccine and therapeutic strategies [18,19].

The incidence of SARS-CoV-2 infections has changed dramatically since early 2020 and these changes have been driven by outbreaks linked to viral genetic variants [13,20]. Thus, during this pandemic, variants of SARS-CoV-2, characteri zed by specific combinations of mutations in their genomes, emerged and spread throughout the world [13,20,21]. They have been characterized by different speeds of propagation, duration of persistence and sensitivity to the antibodies elicited by vaccines or infections. Some of them had a pandemic spread, while others had a more limited expansion.

Almost all SARS-CoV-2 genomic data reported so far are consensus sequences. These do not take into account intra-host genetic diversity, and therefore provide a proxy of the structure of the whole viral population but no information on the presence of minority populations of genomes. The concept of quasispecies was developed during the 1970s by M. Eigen and P. Schuster and describes the clouds of mutants comprised of genetically-linked genomes generated by the accumulation of mutations and possible genetic rearrangements during the replication of RNA viruses [22,23,24,25,26]. Viral quasispecies are generated by genetic variability and are assessed at the intra-host and/or intra-specimen levels. They collectively contribute to the characteristics of the whole viral population and can interact between each other at a functional level and compete with each other, and they are subject to selective pressure [27]. Studying only consensus SARS-CoV-2 genome sequences obtained from respiratory samples of infected patients limits the comprehensive knowledge and understanding of the evolutionary pathways of the virus because some viral quasispecies may expand significantly and cause viral immunological leakage, antiviral drug resistance, and failure of molecular diagnostic tests. The early detection of some minority quasispecies may warn about the possibility of their emergence as majority quasispecies and may predict it. This has been, for instance, observed for HIV drug resistance testing with the early recognition of drug-resistant genomes at a stage they were minority quasispecies [28].

Here, we investigated intra-clinical sample quasispecies of SARS-CoV-2 based on direct next-generation sequencing (NGS) of complete viral genomes in absence of prior PCR amplification, for different viral mutants and variants detected in the clinical virology laboratory of our infectious diseases institute during the first year of the SARS-CoV-2 pandemic.

## 2. Results

### 2.1. Quality and Lineages of the SARS-CoV-2 Genomes Analyzed

Among the 310 sets of SARS-CoV-2 NGS reads that were primarily analyzed, all obtained with the Illumina MiSeq procedure in absence of PCR amplification before sequencing, 110 that were generated from respiratory samples collected from different patients were selected based on their quality. They were characterized by a mean depth of 169 NGS reads per nucleotide position at the genome level. Mean minimal number of reads per nucleotide position was 17 at the genome level (from nucleotides 130 to 29,800) with 3217 positions (11% of the genome) being covered by less than 50 reads per nucleotide position. Mean (± standard deviation) of the maximum number of reads per nucleotide positions in genomes was 594 ± 91. These sets of NGS reads were further processed to analyze the intra- and inter-sample genomic diversities and the SARS-CoV-2 viral quasispecies.

**Figure 1:**
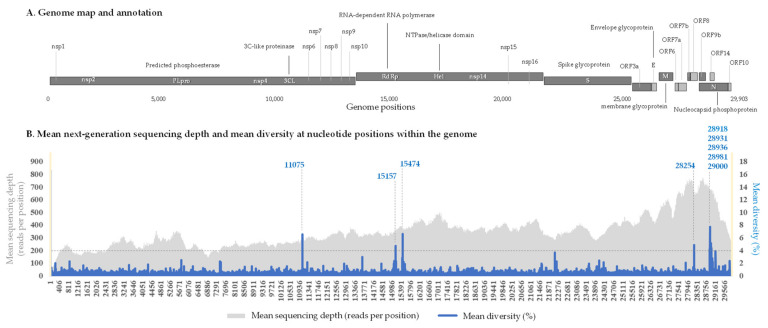
Intra-sample nucleotide diversity along the SARS-CoV-2 genome and hot spots of diversity. (**A**) Genome map and annotation. (**B**) Mean next-generation sequencing depth (in number of reads per nucleotide position) and mean intra-sample genetic diversity at nucleotide positions within the genome (%). Genome positions 1 to 130 and 29800 to 29903 are marked by yellow areas due to their sequencing depth lower than the threshold used in the present analyses. Hot spots of intra-sample genetic diversity are indicated by vertical dashed lines.

The 110 genomes had been obtained from respiratory samples collected between March 2020 and February 2021 (one year). They were classified in various lineages according to the Nextclade [10,29], Pangolin [30], and World Health Organization (WHO) (https://www.who.int/en/activities/tracking-SARS-CoV-2-variants/ (accessed on 3 October 2022)) classifications. A total of 37 genomes were obtained from respiratory samples collected during the first wave in France from patients diagnosed with SARS-CoV-2- between February and May 2020; they included 22 genomes of Nextstrain lineage 20A; 9 of lineage 20B, and 6 of lineage 20C (Supplementary Table S1). In addition, 66 viral genomes were classified in lineages that we named variants Marseille-1 to Marseille-10. These variants were comprised of ≥30 genomes with a specific set of ≥5 mutations and were all first detected between June and August 2020 [20]. These 66 genomes included 4 genomes of the Marseille-1 variant (Pangolin lineage B.1.416), 11 genomes of the Marseille-2 variant (B.1.177), 5 genomes of the Marseille-3 variant (B.1), 16 genomes of the Marseille-4 variant (B.1.160), 9 genomes of the Marseille-5 variant (B.1.367), 3 genomes of the Marseille-6 variant (B.1), 4 genomes of the Marseille-7 variant (B.1.416.1), 5 genomes of the Marseille-8 variant (B.1.1.269), 4 genomes of the Marseille-9 variant (B.1.1.241), and 5 genomes of the Marseille-10 variant (B.1.221). Finally, 6 genomes were classified as of the Alpha variant (B.1.1.7) and one was classified as of the Beta variant (B.1.351). For these 110 genomes, the mean intra-sample genetic diversity at nucleotide positions within the genome (in %) as well as the mean next-generation sequencing depth (in number of reads per nucleotide position) were plotted in Figure 1.

### 2.2. Nucleotide Diversity in the SARS-CoV-2 Genomes and Genes

Mean nucleotide diversity in the 110 selected genomes was 0.21 ± 0.65%. A total of 5357 nucleotide positions with a diversity >4% were identified in the genomes, which represented 17.9% of all nucleotide positions. Among them, 3627 (12.1%) were specific of a given genome and 1730 (5.8%) were shared by different (at least two) genomes. An uneven distribution of nucleotide diversity was observed, as it affected all the genes but at various levels. The mean nucleotide diversity at the SARS-CoV-2 gene level ranged between 0.2 and 0.5% for all samples except for one Marseille-1 genome for which mean gene diversity was 0.9%. Assessment of the number of nucleotide positions exhibiting significant diversity for each gene as a function of its corresponding length revealed that the ORF10 gene had the highest number of positions exhibiting a significant diversity, 0.56 per 100 nucleotide positions (corresponding to a mean genetic diversity of 0.20%), followed by the spike gene exhibiting a mean diversity at 0.34 per 100 nucleotide positions (mean genetic diversity of 0.22%), and the N gene exhibiting a mean diversity at 0.24 per 100 nucleotide positions (mean genetic diversity of 0.34%) (Table 1).

### 2.3. Hot Spots of Intra-Sample Genetic Diversity

The analysis of the mean intra-sample genetic diversity per nucleotide position for the 110 genomes allowed us to identify nine hot spots of diversity (Figure 1). These positions were characterized by a mean intra-sample genetic diversity >4% detected in >45% of the genomes analyzed, considering a mean NGS depth >50 reads/nucleotide position. These hot spots of intra-sample genetic diversity were distributed in three SARS-CoV-2 genes: ORF1ab, ORF8, and N. ORF1ab harbored three hot spots: one in NSP6 that was a U > C substitution or a deletion at position 11075 (mean ± standard deviation of diversity, 6.6 ± 4.3% (range: 0.0–23.4%)); and two positions in NSP12 that were an A > U substitution or a deletion at position 15175 (4.6 ± 2.5% (0.0–11.4%)), and a U > G substitution or a deletion at position 15474 (6.7 ± 3.2% (0.0–15.5%)). In the ORF8 gene, there was a single hot spot of intra-sample genetic diversity, with a deletion of nucleotide A28254 (4.8 ± 4.3% (0.0–17.4%)) that was located close to the end of this gene at position 28259 and generated a frameshift. Finally, we detected five hot spots of intra-sample genetic diversity in the N gene: at positions 28918 (U > G; mean ± standard deviation diversity: 4.3 ± 2.3% (0.0–10.9%)); 28931 (G > U; 4.2 ± 2.4% (0.0–12.7%)); 28936 (G > U; 7.7 ± 3.2% (0.0–14.5%)); 28981 (U > G; 5.2 ± 2.5% (0.0–12.8%)); and 29000 (G > A; 4.4 ± 2.3% (0.0–10.8%)).

In the NSP6 gene that encodes a seven-helix transmembrane protein, substitution U11075C and deletion at position 11075 have been described previously by Kuipers et al. [31] in their study of within-patient genetic diversity of SARS-CoV-2 conducted across a cohort of 4688 sequenced samples collected in 2020 including 749 from Switzerland [31]. Substitution U11075C results in the replacement of a phenylalanine by a leucine at amino acid position 35 of the NSP6 protein, while deletion U11075- introduces a premature stop codon that results in protein truncation. According to the CoV-Spectrum online tool (https://cov-spectrum.org/ (accessed on 20 August 2022)) [32], for all times and everywhere worldwide, substitution U11075C was found in 4205 genomes, mostly of Omicron BA.2 (28.8%), Alpha (9.3%), and Delta (B.1.617.2, 6.2%) variants, and obtained in USA, United Kingdom, France, and Germany. Regarding deletion U11075-, it was found in 9181 genomes, mostly of Omicron BA.1.1 (19.9%), Delta (AY.44, 11.0%), and Alpha (9.5%) variants; these genomes were mostly obtained in USA and Sweden. Regarding the two hot spots of diversity in the NSP12 gene that encodes the viral RNA-dependent RNA polymerase: substitution A15175U was harbored by only 11 genomes according to the CoV-Spectrum tool, mostly of the Alpha variant and in USA and Spain, while deletion U15175- was found in 25 genomes, mostly of the Alpha variant, and mostly obtained in United Kingdom, and Italy. Synonymous substitution U15474G was harbored by only 25 genomes, mostly of the B.1.398 lineage (which was described to have predominated in Lebanon [33]) and in Belgium (n = 8), and Lebanon (n = 6), while deletion U15474- was found in 47 genomes, mostly of the Omicron BA.2 variant and in Chile and France. Regarding the ORF8 gene for which the encoded protein is suspected to interact with the host immune response including through the IFN pathway [34], deletion A28545- causes a frameshift, and a stop codon four codons forward; consequently, the last two residues of the protein are changed and additional four amino acids are incorporated in the C-terminal region of the encoded protein. It was deemed that this mutation most likely did not affect ORF8 activity as modifications were in the C-terminal and non-conserved region of the protein [35]. CoV-Spectrum identified this deletion in 64 genomes, mostly of the Omicron BA.1.1 and Delta (AY.122) variants and in USA, India, Italy, and Chile. Finally, synonymous substitutions U28918G and U28981G and non-synonymous substitutions G28931U (Ala220Ser), G28936U (Leu221Phe), and G29000A (Gly665Ser) in the N gene that encodes the nucleocapsid were harbored by 31, 9, 1432, 3192, and 2307 genomes, respectively. G28936U was predominantly detected in genomes of Omicron BA.1.1 (18.0%), Omicron BA.2.35 (12.3%), and B.1.620 (discovered in Lithuania [36], 7.7%) lineages, and of the XB recombinant (7.3%); these genomes having been mostly obtained in USA, United Kingdom, South Korea, and Germany. G29000A was predominantly detected in genomes of Alpha (22.2%), BA.2.34 (20.1%), BA.2 (8.4%), and BA.1.1 (8.0%) lineages, mostly obtained in USA, United Kingdom, and Norway. The combination of the four nucleotide deletions U11075-, U15175-, U15474-, and A28545- was not harbored by any genomes according to CoV-Spectrum. It is worthy to note that the nine hot spots of diversity revealed here were not identified in a study conducted in China that performed NGS of metatranscriptomic and hybrid captured libraries to characterize intra-host genetic diversity in sequential specimens from eight patients infected during January and February 2020 [37]. This indicates that SARS-CoV-2 quasispecies may vary substantially within time and space.

**Figure 2:**
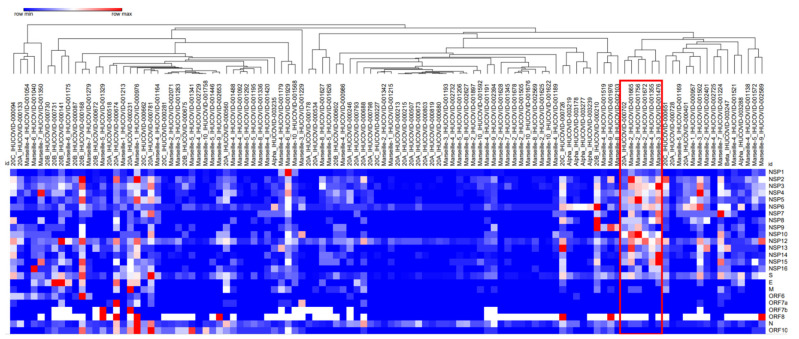
Hierarchical clustering of sets of SARS-CoV-2 next-generation sequencing reads for all 110 samples studied here based on mean intra-sample genetic diversity per 100 nucleotides for each gene. The diversity of all selected positions for this clustering was greater than 4%. The red box indicates a set of six genomes of different lineages with 920 positions exhibiting significant diversity, among which 91 were shared by all the six genomes.

For a global visualization of the numbers of positions exhibiting significant intra-sample genetic diversity for the genomes retrieved from all studied samples, we performed a hierarchical clustering based on the mean intra-sample genetic diversity per 100 nucleotides according to the different genes. This hierarchical clustering allowed us to delineate a group of six genomes of different lineages including three of the Marseille-2 variant; one of each of the Marseille-3 and Marseille-4 variants; and one of the Nextstrain lineage 20A (Figure 2). 

This set of genomes was characterized by a number of 920 positions exhibiting significant diversity, among which 91 were shared by all the six genomes and were distributed in all genes except in the E, ORF6, and ORF7 genes. The NSP3 and the spike genes exhibited the highest number (n = 196) of such positions.

### 2.4. Correlation between Intra-Sample and Inter-Sample Genetic Diversity in SARS-CoV-2 Genomes

**Figure 3:**
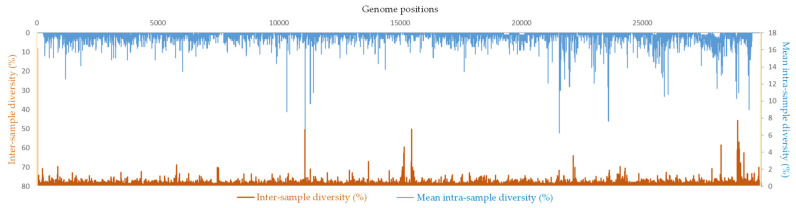
Correlation between the intra-sample genetic diversity in the present study and the inter-patient diversity at the global scale.

To try correlating at a given nucleotide position the intra-sample genetic diversity in the SARS-CoV-2 genomes studied here and the inter-sample and inter-patient diversity at the largest scale, we superimposed the mean intra-sample genetic diversity for the 110 genomes analyzed here and the global diversity at all SARS-CoV-2 nucleotide positions available from the Nextstrain online tool (https://nextstrain.org/ncov/gisaid/global/all-time (accessed on 30 September 2022)). Nucleotide positions were selected if they exhibited an intra-sample diversity >1% and a non-null inter-sample diversity, in order to detect significant concordances. Thus, 118 nucleotide positions (0.4% of all genome positions) were identified that showed a diversity at the intra-sample as well as inter-patient levels. They included 35 positions in ORF1a, 16 positions in ORF1b, 2 positions in ORF3a, 14 positions in S (spike gene), 2 positions in E (envelope gene), 1 position in ORF7b, 5 positions in ORF8, and 43 positions in N (nucleocapsid gene) (Table 2; Figure 3).

When considering intra-sample genetic diversity of at least 4% and a global diversity of at least 1%, six positions were identified, including one in ORF1a (at position 11075 in NSP6), one in ORF8 (at position 28254), and four in the N gene. These six mutations were part of the nine hot spots of intra-sample genetic diversity.

### 2.5. Presence in Viral Quasispecies of Variant Hallmark Mutations of the Spike Gene

The SARS-CoV-2 spike protein is critical for viral entry into the host cell, a major target of immune responses elicited by infection or vaccine immunization, and the vaccine target [38]. Multiple mutations have been observed in the spike gene of SARS-CoV-2 variants along the pandemic, and some have been involved with viral escape [38]. To assess if intra-sample genetic diversity could translate into the emergence of new lineages and variants characterized by specific mutations within spike, we studied the intra-sample genetic diversity of six codons of the spike gene (417, 452, 484, 501, 614, 681) harboring mutations that are markers of major viral variants, specifically the diversity at nucleotide positions implicated by the mutations within these codons: positions 22813 (where G > U causes amino acid substitution K417N), 22917 (U > G: L452R), 23012 (G > A: E484K), 23063 (A > U: N501Y), 23403 (A > G: D614G), and 23604 (C > A: P681H). In our dataset, intra-sample diversity was >4% in a single sample, from which an Alpha variant was identified. Therefore, the overall level of intra-sample genetic diversity was low, the mean (± standard deviation) diversity at the six positions being 0.31 ± 1.96% (range, 0.00–38.80%) and ranging between 0.17 ± 0.63% (range, 0.00–5.10%) and 0.57 ± 1.62% (0.00–13.20%) according to the position. Considering any level of diversity, between 8.2 and 30.9% of the genomes, and 27 of the 110 genomes overall, exhibited a genetic diversity at any of these six positions. The variants Marseille-2 (one genome), Marseille-5 (two genomes), and Alpha (two genomes) exhibited an intra-sample genetic diversity at position 22813 corresponding to mutation G22813U, which leads to amino acid substitution K417N where the U corresponds to the minority nucleotide compared to the consensus. Two genomes of lineage 20A and one genome of the Marseille-1 variant exhibited a diversity at position 23604 corresponding to mutation C23604A, which leads to amino acid substitution P681H, where the A corresponds to the minority nucleotide compared to the consensus. Otherwise, significant intra-sample genetic diversity was observed at position 22813 in six genomes of the Marseille-2 (n = 2 genomes), Marseille-4 (n = 3), and Marseille-5 (n = 1) variants; at position 23012 in 10 genomes of lineages 20A (n = 2) and 20C (n = 1) and of the Marseille-1 (n = 1), Marseille-2 (n = 3), Marseille-3 (n = 2), and Marseille-4 (n = 1) variants; at position 23063 in 14 genomes of lineages 20A (n = 4 genomes) and 20B (n = 1), and in the Marseille-2 (n = 2), Marseille-3 (n = 1), Marseille-4 (n = 1), Marseille-5 (n = 1), Marseille-9 (n = 1), Marseille-10 (n = 1) and Alpha (n = 2) variants; at position 23403 in 34 genomes of lineages 20A (n = 6 genomes), 20B (n = 4), and 20C (n = 3), and in the Marseille-1 (n = 2), Marseille-2 (n = 2), Marseille-3 (n = 3), Marseille-4 (n = 5), Marseille-5 (n = 5), Marseille-10 (n = 1) and Alpha (n = 3) variants; at position 23604 in 14 genomes of lineages 20A (n = 3 genomes), 20B (n = 1), and 20C (n = 2), and in the Marseille-1 (n = 1), Marseille-2 (n = 1), Marseille-4 (n = 2), Marseille-8 (n = 1), and Alpha (n = 3) variants. Finally, the mean proportions of genomes exhibiting an intra-sample genetic diversity at these six positions were greater than 10% for 7 of the 15 lineages or variants studied here, including the lineages 20A and 20C and the variants Marseille-1, Marseille-2, Marseille-3, Marseille-4, and Alpha.

### 2.6. Intra-Sample Genetic Diversity for the Different SARS-CoV-2 Lineages and Variants

Finally, the hierarchical clustering performed based on the levels of intra-sample genetic diversity per genome and gene showed that genomes from same lineages or variants were not clustered together but were often scattered in different clusters regardless of their classification (Figure 2). The same observation was made when taking only into account nucleotide positions of the spike gene. Thus, we were unable to identify patterns of intra-sample genetic diversity that were specific to a given lineage or variant. Moreover, we found that the mean intra-sample genetic diversity per nucleotide position was 0.50% for the Marseille-1 variant; 0.30% for the Marseille-3 variant; 0.20% for the lineages 20A, 20B, and 20C, and for the Marseille-2, Marseille-4, Marseille-5, Marseille-7, Marseille-8, Marseille-9, and Alpha variants; and 0.10% for the Marseille-6, Marseille-7, and Beta variants. Additionally, the mean (± standard deviation) proportions of nucleotide positions exhibiting a significant intra-sample diversity ranged between 0.14 ± 0.10% (range, 0.04–0.29%) for the Marseille-10 genomes and 0.57 ± 0.45% (0.12–1.10%) for the Marseille-1 genomes. These mean proportions were 0.32 ± 0.32% (range, 0.05–1.15%), 0.43 ± 0.0.34% (range, 0.10–0.88%), and 0.51 ± 0.38% (range, 0.13–1.28%) for the genomes of lineages 20A, 20C, and 20B, respectively, and 0.22 ± 0.11% (0.09–0.40%), 0.22 ± 0.14% (0.03–0.59%), and 0.31 ± 0.33% (0.04–0.92%) for the genomes of Alpha, Marseille-4, and Marseille-2 variants, respectively.

## 3. Discussion

We investigated here the SARS-CoV-2 quasispecies recovered by NGS directly from nasopharyngeal samples for 110 genomes covering all major lineages that circulated in France during the first year after first detection of SARS-CoV-2 in our geographical area. For this, only genomes with a coverage greater than 99% of the genome GenBank Accession no. NC_045512.2 and a mean NGS depth greater than 50 reads/nucleotide position were selected for the present analyses. These genomes were classified in lineages 20A, 20B, and 20C that circulated during early 2020 as well as in several variants that were detected in our clinical virology laboratory during summer 2020, among which were the Marseille-1 variant that had a limited spread and was reported to originate from Northern Africa [39], the Marseille-2 variant that predominated in Spain [40], and the Marseille-4 variant that predominated in France [41].

In most studies on SARS-CoV-2 quasispecies, NGS of viral genomes has been performed after a step of multiplex PCR amplification with a set of SARS-CoV-2 specific oligonucleotide primers, in most cases according to the so-called ARTIC procedure that is widely used in research and for genomic epidemiology [42,43]. This step allows obtaining SARS-CoV-2 genomes from clinical samples with lower viral loads by generating overlapping amplicons covering the whole genome sequence. However, prior PCR amplification can introduce quantitative and qualitative sequencing biases by favoring some genetic populations while neglecting others. Such biases in determining intra-sample viral diversity have been reported for various viruses, among which are HIV, hepatitis C virus, and SARS-CoV-2 [43,44,45,46]. Here, NGS of SARS-CoV-2 genomes had been performed in absence of prior multiplex PCR amplification, which limited the sequencing biases of viral quasispecies and may have contributed to their more accurate assessment. In return, this led to far lower sequencing depths than those obtained when using the ARTIC procedure, which may have impaired the detection of very minority viral quasispecies and is a limit of the present study.

We identified in this study that approximately one-fifth of nucleotide positions in the SARS-CoV-2 genomes showed intra-sample genetic diversity including in at least two viral genomes in about one-third of these cases, and in a single genome in two-thirds of the cases. This revealed the presence of substantial amounts of SARS-CoV-2 quasispecies in our dataset and delineated pan and core sets of nucleotide positions with intra-sample genetic diversity at the genome level. Although this diversity was scattered along the genomes, it was unevenly distributed in viral genes. The genes with the greatest diversity per 100 nucleotide positions were the ORF10, spike (S), and nucleocapsid (N) genes. These three genes were described in a previous study of 94 clinical samples from 48 patients in China as among those with the highest diversity, with proportions of positions exhibiting diversity per 1000 nucleotides that were >10% for ORF10, between 5–10% for the N gene, and about 5% for the S gene [47]. ORF10 is a short (38-amino-acid-long) accessory protein for which the protein-protein interaction map suggested it may modulate the cellular ubiquitination or palmitoylation system to facilitate viral replication [3]. Recently, it was reported that its overexpression facilitated viral infection by blocking STING-induced IFN production and autophagy, hence viral immune evasion [48]). The SARS-CoV-2 spike protein is 1273 amino acids long, interacts with the host cell receptor ACE2, and allows the virus entry into host cells [3]. It is highly glycosylated and a major target of neutralizing antibodies, which led to its use for the design of most vaccines [38]. Regarding the nucleocapsid protein, it is 419 amino acids long and packages the viral RNA to form a ribonucleocapsid [4]. 

We observed nine hot spots of intra-sample diversity in the SARS-CoV-2 genomes, of which five corresponded to non-synonymous mutations. These nine hot spots were located in the NSP6, NSP12, ORF8, and N genes. Two of these hot spots were previously described. Kuipers et al. [31] studied 3939 deeply sequenced genomes and found that the most diverse nucleotide position, with mutations in about half of the samples, was position 11075 in the NSP6 gene [31]. As we observed here, the diversity consisted of a deletion or a U > C substitution. Gaurav et al. [35] described deletion A28545- observed here in SARS-CoV-2 from India [35]. Beyond, nucleotide diversity at several of these hot spots corresponded to mutations that were encountered in SARS-CoV-2 consensus genomes obtained worldwide, as assessed by the CoV-Spectrum tool. Finally, approximately 1 out of 2500 genome positions were identified that showed intra-sample diversity in the present work and concurrently inter-sample diversity between genomes obtained from samples collected worldwide as measured by Nextstrain (https://nextstrain.org/ncov/gisaid/global/all-time (accessed on 30 September 2022)) [10]. Moreover, a set of six genomes among those analyzed here was identified by hierarchical clustering that was characterized by a set of 920 positions exhibiting intra-sample genetic diversity, among which 91 were shared by these six genomes. This intra-sample diversity was distributed in all but three of the SARS-CoV-2 genes. These six genomes were classified as belonging to different SARS-CoV-2 variants.

Taken together, these findings suggest that the intra-sample genetic diversity observed through SARS-CoV-2 quasispecies is not random but is shaped by fitness advantage and positive selection and argue for its epidemiological and biological significance. Armero et al. [49] reported similar findings, with one quarter of the nucleotides exhibiting intra-sample diversity in SARS-CoV-2 genes ORF1a, ORF1b, S, and N, which were shared among 210 clinical specimens collected between January and April of 2020 in Australia and analyzed by NGS, suggesting host-to-host transmission [49]. As a matter of fact, SARS-CoV-2 quasispecies may represent a pool of mutations for epidemic mutants and variants as their dynamic enhances the likelihood of selection of viral mutants or variants with enhanced capabilities of replication and of overcoming selective constraints [50]. Hence, viral intra-host diversity reflects the mutational patterns that have the potential to emerge and will eventually spread at a broad scale among SARS-CoV-2-infected patients. In a previous study, Quaranta et al. [51] reported intra-host evolution over a period of 109 days with the emergence of 26 amino acid mutations and two deletions, of which 57% were in the spike gene [51]. Additionally, Chaguza et al. [52] reported an approximately two-fold accelerated SARS-CoV-2 intra-host evolution during chronic infection over a period of 471 days that led to the emergence of distinct genotypes [52]. Choi et al. [53] reported the SARS-CoV-2 infection over a period of 151 days in an immunocompromised patient who received remdesivir as well as an anti-spike antibody cocktail, and in whom quasispecies analysis showed the occurrence of 12 non-synonymous substitutions in the spike gene, among which substitutions N501Y ± E484K present in several variants of concern including the Alpha, Beta, Gamma, Delta, and Omicron variants [53]. Additionally, Vellas et al. reported the emergence of spike mutations E484K or Q493K among viral quasispecies in 5 of 23 patients treated with the combination of monoclonal antibodies Bamlanivimab/Etesevimab [54].

Here, the comparison of intra-sample genetic diversity according to viral genomes classification did not show sharp variant-specific signatures, neither at the level of intra-sample diversity at the gene level for whole genomes nor at spike gene nucleotide positions. This contrasts with a previous study that reported the specific detection of intra-host diversity at some positions in some lineages [49]. Beyond, we did not observe an increasing trend over time of the mean proportions of nucleotide positions exhibiting significant diversity in our dataset as, regarding these proportions, samples collected during the early pandemic from which lineages 20A, 20B, and 20C were retrieved were intermixed with samples collected since summer 2020 and from which various variants were retrieved. This could suggest that the increase of the number of mutations accumulated in SARS-CoV-2 genome over time, which is approximately two mutations per month [9], was not combined with a greater intra-sample diversity, but this deserves to be further assessed in several other studies with larger datasets.

We have been attentive in the present study to the intra-sample diversity at spike nucleotide positions where mutations have been reported to occur that are hallmarks of SARS-CoV-2 variants of concern, and that are critical for immune escape and were reported to emerge during prolonged infection of immunocompromised patients, at codons 417, 452, 484, 501, 614, and 681 [38]. Amino acid substitution K417N is a hallmark of the Omicron variants, occurs in a neutralizing epitope, and has been suspected to be associated with escape from neutralization by some classes of monoclonal antibodies and convalescent patients’ serum samples, and to contribute to escape from neutralization by antibodies elicited by mRNA vaccines [55]. Amino acid substitution L452R is a hallmark of the Delta variant but also occurred independently in several other variants, indicating convergent evolution, suggesting that this amino acid substitution could result in viral adaptation due to increasing immunity at the population scale, and it has been shown to reduce neutralization by several monoclonal antibodies and convalescent patients’ plasma [56]. Amino acid substitution E484K is located in the receptor binding domain of the spike protein, enhances binding affinity to ACE2, and is also located in a major virus neutralization site and decreases binding affinity of some neutralizing antibodies [55]. Another amino acid substitution at this position, E484Q, which could weaken virus binding to ACE2, is a hallmark of the Delta variant (https://covariants.org (accessed on 30 September 2022)) [40,55]. Amino acid substitution N501Y is located within the receptor binding motif of the spike S1 subunit and enhances virus binding affinity to the host cell. It has been a hallmark mutation of several variants including the Alpha, Beta, Gamma, and Omicron variants (https://covariants.org (accessed on 30 September 2022)) [40,55]. Amino acid substitution D614G favors an open conformational state for the spike and was associated with increased infectiousness in vitro, with increased viral loads in the upper airways, and was suspected to be associated with a higher rate of profitable binding with the host receptor [57,58]. Amino acid substitution P681H [59] is in the cleavage site of the spike subunits S1/S2 and is predicted to increase cleavage by furin, potentially impacting the viral cell entry. In our dataset, we observed a diversity at ≥1 of these positions in one quarter of the 110 genomes, although most often at a low level, below the threshold of 4% that was used for significance.

Finally, it is worthy to note that it was reported that the SARS-CoV-2 quasispecies differed in the same patient according to the clinical samples in which they were investigated, which is another hint of the existence of significant bottlenecks and selection processes for these viral quasispecies [9,60]. Moreover, the quasispecies complexity of sputum samples was reported to be significantly lower than that of nasopharyngeal swabs [50]. Therefore, the spectrum of viral quasispecies present in a given patient will differ according to the sample and to time of sampling.

In summary, the present study revealed a pan set and a core set of nucleotide positions exhibiting intra-sample genetic diversity among studied nasopharyngeal samples that were found to be infected with members of the SARS-CoV-2 lineages that predominated during the first year of the pandemic. It illustrates that the SARS-CoV-2 consensus genome sequences are only incomplete and imperfect representations of the entire viral populations infecting a patient. A substantial diversity of viruses is present in a same patient that can be submitted to bottlenecks and selective pressures. The existence of hot spots of intra-sample genetic diversity suggests that at least some of these mutations are not only de novo mutations generated by the low fidelity RNA-dependent RNA polymerase and might be selected and found in major viral variants. In this view, Zhang et al. reported that some of the minority SARS-CoV-2 quasispecies that were detectable during the early stage of the pandemic did forecast later circulating mutants and variants [5,6]. Thus, studies of viral quasispecies as the present one may reveal nucleotide positions in the SARS-CoV-2 genome that particularly exhibit genetic diversity and variability, and hence possible virus evolutionary pathways and critical genomic regions for the virus. These are important observations to understand the associations of viral genetic patterns with the spread, transmissibility, and pathogenicity of novel viral lineages, and can provide hints of putative targets for the development of therapeutics. Finally, it is worthy to note that although the analyses of SARS-CoV-2 quasispecies allow a tremendously finer characterization of minority genomes than the analyses of consensus genome sequences, still, they may be biased. Indeed, quasispecies that are a very small minority at a given time point may be differently detected according to the whole NGS process used [43,45,46], and those with impaired replicative capabilities due to mutations can remain undetected whatever the NGS protocol used. Nonetheless, previous data strongly argue to consider more systematically, retrospectively, and prospectively in future studies the analysis of SARS-CoV-2 quasispecies among NGS reads generated from clinical samples to more accurately decipher the SARS-CoV-2 evolutionary pathways.

## 4. Materials and Methods

### 4.1. Next-Generation SARS-CoV-2 Genome Sequencing Methods and Data

The data generated by NGS of SARS-CoV-2 genomes analyzed here had been obtained in the framework of SARS-CoV-2 genotyping performed since the first diagnosis of SARS-CoV-2 performed by real-time reverse transcription-PCR (qPCR) during late February 2020 in our clinical microbiology–virology laboratory at university hospital institute (IHU) Méditerranée Infection in university hospitals of Marseille, southeastern France, as previously reported [20]. These SARS-CoV-2 genomes are available from the NCBI GenBank nucleotide sequence database (https://www.ncbi.nlm.nih.gov/genbank/ (accessed on 30 September 2022)) [61] with no. OP646492-OP646601, and from the GISAID sequence database (https://www.gisaid.org/ (accessed on 30 September 2022)) [62] using the GISAID online search tool with “IHU” and “France” as keywords or the correspondence table between GenBank and GISAID identifiers provided as Supplementary Table S2.

Raw NGS data used in the present analyses were those generated from a total of 310 nasopharyngeal samples collected from SARS-CoV-2-positive patients between March 2020 and February 2021 and directly sequenced without prior PCR amplification by the Illumina technology with the Nextera XT paired-end strategy on a MiSeq instrument (Illumina Inc., San Diego, CA, USA), as previously reported [20]. Reads obtained had been mapped on the SARS-CoV-2 complete genome of the Wuhan-Hu-1 isolate (GenBank Accession no. NC_045512.2) with the CLC genomics workbench software v7 using the following thresholds: 0.8 for sequence coverage and 0.9 for nucleotide similarity (https://digitalinsights.qiagen.com/ (accessed on 30 September 2022)). Only the files corresponding to complete genomes with a coverage of at least 99% of the genome no. NC_045512.2 were taken into consideration for further analyses. The SARS-CoV-2 genotype had been identified on the basis of the consensus genome sequence with the Nextclade tool v1.6.0 (https://clades.nextstrain.org (accessed on 30 September 2022)) [29].

### 4.2. Detection and Characterization of Genetic Quasispecies

The complete genome mapping data generated by the CLC software were exported from the mapping output file as tab separated values (.tsv) files. In these latter files, nucleotides were detected, and corresponding numbers of reads were mentioned for each nucleotide position of the assembled genomes. These data were automatically analyzed using an in-house tool created through the Microsoft Excel software (https://www.microsoft.com/fr-FR/microsoft-365/excel (accessed on 30 September 2022)) that we named “QuasiS”. QuasiS allows superimposing the consensus genome sequence obtained to the Wuhan-Hu-1 isolate genome GenBank Accession no. NC_045512.2. It further calculates for each position of the genome the intra-sample nucleotide diversity, which is the proportion of reads covering a given position that does not harbor the consensus nucleotide. In addition, it indicates whether the consensus nucleotides differ from those harbored by the reference genome and calculates the mean, standard deviation, and minimum and maximum values for diversity for the whole genome and the proportion of nucleotide positions covered with more than 50 reads. The positions of the different viral genes are indicated in the QuasiS tool, and genes can be selected and scrutinized individually. Alarms are generated in case of diversity above a defined value, and contiguous positions with such level of diversity are also indicated. Finally, nucleotide diversity and genome coverage by NGS reads are plotted into separated graphics. Based on QuasiS files generated for each clinical sample, the numbers of reads per nucleotide position were used to guide the selection of NGS data to be subsequently characterized, considering only genomes with a mean number of reads per position higher than 50 and a coverage greater than 99% of the genome GenBank Accession no. NC_045512.2.

### 4.3. Analysis of SARS-CoV-2 Intra-Sample Genetic Diversity at the Genome and Gene Scales

An intra-sample nucleotide diversity of 4% at a given nucleotide position was defined as a threshold for a significant diversity, on the basis that this corresponds to at least two reads for a minimum of 50 reads per position, and as we only analyzed genomes for which the mean number of reads per position was higher than 50. For the selected set of SARS-CoV-2 genomes, the mean intra-sample genetic diversity at nucleotide positions within the genome (in %) and the mean NGS depth (in number of reads per nucleotide position) were both plotted through the Microsoft Excel software. To study the correlation between genetic diversity at nucleotide positions either intra-sample or inter-samples, we compared intra-sample diversity in the genomes studied here and genetic diversity at the largest available scale that was recovered as a .tsv file from the Nextstrain website (https://nextstrain.org/ncov/gisaid/global/all-time (accessed on 30 September 2022)) [10] that displays genomic epidemiology of SARS-CoV-2. The correlation between the intra-sample genetic diversity in the present study and the inter-patient diversity at the global scale was represented as a graph generated with the Microsoft Excel software. Genome positions were identified as exhibiting nucleotide diversity and were selected if they exhibited an intra-sample diversity >1% and a non-null inter-sample diversity, to detect significant concordances. For a global visualization of the mean intra-sample genetic diversity and of the number of variable positions for all the samples, we performed a hierarchical clustering using the Morpheus web application (https://software.broadinstitute.org/morpheus/ (accessed on 30 September 2022)) [63].

The frequencies of nucleotide mutations within SARS-CoV-2 genomes as well as the countries where they were detected were retrieved from the CoV-Spectrum online tool (https://cov-spectrum.org/ (accessed on 20 August 2022)) [32]. CoV-Spectrum allowed searching the mutation in 12,922,519 genomes collected worldwide between 6 January 2020 and 20 August 2022.

## Figures and Tables

**Table 1:** Intra-sample diversity per gene as a function of its corresponding length.

Genes	Coordinates on the Genome GenBank Accession no. NC_045512.2	Mean Diversity (%)	Number of Gene Positions Exhibiting a Significant (>4%) Diversity in Any of the 110 Samples	Number of Positions per 100 Nucleotides
ORF1ab	266..21555	0.2	3123	0.15
S	21563..25384	0.22	1281	0.34
ORF3a	25393..26220	0.2	105	0.13
E	26245..26472	0.2	31	0.14
M	26523..27191	0.21	68	0.10
ORF6	27202..27387	0.17	26	0.14
ORF7a	27394..27759	0.2	28	0.08
ORF7b	27756..27887	0.19	4	0.03
ORF8	27894..28259	0.21	14	0.04
N	28274..29533	0.34	301	0.24
ORF10	29558..29674	0.2	65	0.56

**Table 2:** Nucleotide positions within SARS-CoV-2 genomes exhibiting intra-sample and inter-patient diversity.

Coordinates on the Genome GenBank Accession no. NC_045512.2	Gene_Codon	Inter-Patient Diversity (%)	Mean Intra-Sample Diversity (%)	Nucleotide Position in Codon
516	ORF1a_84	11	1.21	1
517		11	1.06	2
518	ORF1a_85	13	1.25	1
519		13	1.11	2
520		13	1.15	3
521	ORF1a_86	6	1.03	1
522		6	1.27	2
867	ORF1a_201	1	1.01	2
868		1	2.33	3
873	ORF1a_203	4	1.41	2
963	ORF1a_233	1	1.12	2
1465	ORF1a_400	3	1.61	3
1600	ORF1a_445	7	1.05	3
3067	ORF1a_934	1	1.12	3
3468	ORF1a_1068	2	1.65	2
4318	ORF1a_1351	1	1.76	3
5434	ORF1a_1723	1	1.03	3
5743	ORF1a_1826	1	1.24	3
5886	ORF1a_1874	1	1.50	2
6268	ORF1a_2001	2	1.51	3
6713	ORF1a_2149	2	1.24	3
9044	ORF1a_2927	1	1.10	1
9714	ORF1a_3150	1	1.47	2
10037	ORF1a_3258	3	1.23	1
11075	ORF1a_3604	1	6.64	1
11117	ORF1a_3618	5	1.02	1
11289	ORF1a_3675	32	1.63	2
11290		32	1.57	3
11291	ORF1a_3676	37	1.57	1
11292		37	1.68	2
11293		37	2.03	3
11294	ORF1a_3677	37	1.73	1
11295		37	1.67	2
11296		37	1.72	3
11997	ORF1a_3911	1	1.57	2
15156	ORF1b_572	1	1.14	2
15157		1	3.87	3
15173	ORF1b_578	1	2.18	1
15491	ORF1b_684	1	1.52	1
15492		1	1.01	2
15500	ORF1b_687	1	1.15	1
15501		1	1.11	2
15576	ORF1b_712	1	1.11	2
17152	ORF1b_1237	1	1.34	3
17514	ORF1b_1358	1	1.01	2
18314	ORF1b_1625	1	1.14	1
18354	ORF1b_1638	2	1.21	2
18482	ORF1b_1681	6	1.25	1
19477	ORF1b_2012	2	1.31	3
21243	ORF1b_2601	1	1.05	2
21492	ORF1b_2684	1	1.23	2
21765	S_68	4	1.19	2
22214	S_218	3	2.25	1
22216		3	1.13	3
22218	S_219	1	1.38	2
22219		1	1.15	3
22645	S_361	1	1.03	3
23531	S_657	3	1.18	1
23534	S_658	1	1.09	1
23622	S_687	2	1.16	2
23642	S_694	3	1.88	1
23652	S_697	1	1.14	2
24038	S_826	2	1.20	1
24089	S_843	1	2.30	1
24365	S_935	1	1.29	1
25620	ORF3a_76	2	1.26	3
25979	ORF3a_196	3	1.66	2
26390	E_49	2	1.01	2
26433	E_63	1	1.31	3
27870	ORF7b_37	5	2.10	3
28215	ORF8_108	1	1.38	1
28251	ORF8_120	11	1.15	1
28252	ORF8_120	11	1.01	2
28253		11	1.17	3
28254	ORF8_121	22	4.84	1
20918	N_215	4	4.27	3
28920	N_216	2	2.70	2
28921		2	1.24	3
28922	N_217	2	1.10	1
28923		2	1.69	2
28924		2	2.42	3
28926	N_218	1	1.52	2
28927		1	3.04	3
28929	N_219	1	1.87	2
28931	N_220	5	4.20	1
28933		5	1.23	3
28949	N_226	1	1.12	1
28952	N_227	1	1.56	1
28954		1	2.84	3
28959	N_229	3	1.49	2
28962	N_230	1	1.77	2
28967	N_232	5	1.31	1
28974	N_234	31	1.92	2
28976	N_235	6	1.07	1
28979	N_236	1	1.33	1
28981		1	5.20	3
28985	N_238	7	2.52	1
28987		7	1.46	3
28989	N_239	1	2.27	2
28994	N_241	1	1.96	1
28997	N_242	1	2.36	1
29000	N_243	3	4.36	3
29004	N_244	3	1.45	2
29010	N_246	1	1.11	2
29014	N_247	1	1.08	3
29021	N_250	2	1.62	1
29024	N_251	1	1.12	1
29029	N_252	4	1.93	3
29035	N_254	2	1.42	3
29039	N_256	2	1.69	1
29041		2	2.43	3
29049	N_259	1	2.77	2
29057	N_262	1	1.55	1
29059		1	2.23	3
29072	N_267	1	1.13	1
29325	N_351	1	1.49	2
29336	N_355	1	1.37	1
29514	N_414	7	1.25	2

## Data Availability

Viral genomes analyzed in the present study are available from the NCBI GenBank nucleotide sequence database (https://www.ncbi.nlm.nih.gov/genbank/ (accessed on 30 September 2022)) [61] with no. OP646492-OP646601, and from the GISAID sequence database (https://www.gisaid.org/ (accessed on 30 September 2022)) [62] using the GISAID online search tool with “IHU” and “France” as keywords and correspondence from Supplementary Table S1.

## Supplementary Materials

The supporting information can be downloaded at: https://www.mdpi.com/article/10.3390/ijms232415658/s1.
