# Supplementary material for: Quasispecies Analysis of SARS-CoV-2 of 15 Different Lineages during the First Year of the Pandemic Prompts Scratching under the Surface of Consensus Genome Sequences
Source: Int J Mol Sci. 2022 Dec 10;23(24):15658. doi: 10.3390/ijms232415658 (PMC9779826; doi:10.3390/ijms232415658)
Supplement: Supplementary file 1 [file ijms-23-15658-s001.zip › ijms-2057980-supplementary.pdf]

Supplementary Materials for:

Quasispecies Analysis of SARS-CoV-2 of 15 Different Lineages during the First Year of the Pandemic Prompts Scratching under the Surface of Consensus Genome Sequences

Wahiba Bader [1,2], Jeremy Delerce [1], Sarah Aherfi [1,2,3], Bernard La Scola [1,2,3] and Philippe Colson [1,2,3,*]

[1] IHU Méditerranée Infection, 19–21 Boulevard Jean Moulin, 13005 Marseille, France; wahibabader.com@gmail.com (W.B.); jeremy.delerce@univ-amu.fr (J.D.); sarah.aherfi@ap-hm.fr (S.A.); bernard.la-scola@univ-amu.fr (B.L.S.)
[2] Microbes Evolution Phylogeny and Infections (MEPHI), Institut de Recherche pour le Développement (IRD), Aix-Marseille University, 27 Boulevard Jean Moulin, 13005 Marseille, France
[3] Assistance Publique-Hôpitaux de Marseille (AP-HM), 264 rue Saint-Pierre, 13005 Marseille, France
* Correspondence: philippe.colson@univ-amu.fr; Tel.: +33-413-732-024

Supplementary Table S1. Number and classification of the genomes analyzed in the present study, and their sequencing depth

SARS-CoV-2 lineage	20A	20B	20C	Marseille-1	Marseille-2	Marseille-3	Marseille-4	Marseille-5	Marseille-6	Marseille-7	Marseille-8	Marseille-9	Marseille-10	Alpha	Beta	Total
Total number of respiratory samples	36	13	15	18	36	18	66	41	8	7	15	10	9	17	1	310
Number of selected respiratory samples	22	9	6	4	11	5	16	9	3	4	5	4	5	6	1	110
Mean next-generation sequencing depth	332	106	242	121	240	168	165	140	72	99	223	83	266	205	76	169
Standard deviation of next-generation sequencing depth	110	46	80	62	188	94	81	74	31	39	114	38	244	92	26	88
Minimum next-generation sequencing depth	17	10	11	20	34	13	13	12	6	12	22	7	34	30	10	17
Maximum next-generation sequencing depth	729	353	505	397	1193	654	560	497	225	260	782	267	1502	603	171	580

Supplementary Table S2. GenBank Accession numbers and IHU Méditerranée Infection identifiers of the genomes analyzed in the present study, and collection dates and genotypes

GenBank_ID	Sequence_ID	Collection date	Genotype
OP646492	IHUCOVID-000133	13-Mar-20	20A
OP646493	IHUCOVID-000662	16-Mar-20	20C
OP646494	IHUCOVID-000141	19-Mar-20	20B
OP646495	IHUCOVID-000087	20-Mar-20	20B
OP646496	IHUCOVID-000651	20-Mar-20	20C
OP646497	IHUCOVID-000094	22-Mar-20	20C
OP646498	IHUCOVID-000168	25-Mar-20	20B
OP646499	IHUCOVID-000178	26-Mar-20	20A
OP646500	IHUCOVID-000213	28-Mar-20	20A
OP646501	IHUCOVID-000215	28-Mar-20	20A
OP646502	IHUCOVID-000210	28-Mar-20	20B
OP646503	IHUCOVID-000231	29-Mar-20	20A
OP646504	IHUCOVID-000401	29-Mar-20	20A
OP646505	IHUCOVID-000227	29-Mar-20	20C
OP646506	IHUCOVID-000246	30-Mar-20	20A
OP646507	IHUCOVID-000281	31-Mar-20	20C
OP646508	IHUCOVID-000507	6-Apr-20	20A
OP646509	IHUCOVID-000518	6-Apr-20	20A
OP646510	IHUCOVID-000534	7-Apr-20	20A
OP646511	IHUCOVID-000560	8-Apr-20	20A
OP646512	IHUCOVID-000602	10-Apr-20	20B
OP646513	IHUCOVID-000673	15-Apr-20	20A
OP646514	IHUCOVID-000674	15-Apr-20	20A
OP646515	IHUCOVID-000680	15-Apr-20	20A
OP646516	IHUCOVID-000672	15-Apr-20	20B
OP646517	IHUCOVID-000675	15-Apr-20	20B
OP646518	IHUCOVID-000688	16-Apr-20	20A
OP646519	IHUCOVID-000702	16-Apr-20	20A
OP646520	IHUCOVID-000726	20-Apr-20	20C
OP646521	IHUCOVID-000728	21-Apr-20	20A
OP646522	IHUCOVID-000730	21-Apr-20	20B
OP646523	IHUCOVID-000731	21-Apr-20	20B
OP646524	IHUCOVID-000781	27-Apr-20	20A
OP646525	IHUCOVID-000793	1-May-20	20A
OP646526	IHUCOVID-000798	2-May-20	20A
OP646527	IHUCOVID-000803	5-May-20	20A
OP646528	IHUCOVID-000819	6-May-20	20A
OP646529	IHUCOVID-000957	13-Jul-20	Marseille-1
OP646530	IHUCOVID-000976	23-Jul-20	Marseille-1
OP646531	IHUCOVID-001040	31-Jul-20	Marseille-6
OP646532	IHUCOVID-001054	4-Aug-20	Marseille-4
OP646533	IHUCOVID-001092	5-Aug-20	Marseille-4
OP646534	IHUCOVID-001138	7-Aug-20	Marseille-4
OP646535	IHUCOVID-000986	9-Aug-20	Marseille-4
OP646536	IHUCOVID-001502	10-Aug-20	Marseille-3
OP646537	IHUCOVID-001420	10-Aug-20	Marseille-4

Supplementary Table S2 (continued)

GenBank_ID	Sequence_ID	Collection date	Genotype
OP646538	IHUCOVID-001446	10-Aug-20	Marseille-5
OP646539	IHUCOVID-001476	11-Aug-20	Marseille-3
OP646540	IHUCOVID-001488	11-Aug-20	Marseille-4
OP646541	IHUCOVID-001505	12-Aug-20	Marseille-2
OP646542	IHUCOVID-001521	12-Aug-20	Marseille-4
OP646543	IHUCOVID-001519	12-Aug-20	Marseille-4
OP646544	IHUCOVID-001164	13-Aug-20	Marseille-4
OP646545	IHUCOVID-001169	14-Aug-20	Marseille-5
OP646546	IHUCOVID-001175	14-Aug-20	Marseille-6
OP646547	IHUCOVID-001179	15-Aug-20	Marseille-4
OP646548	IHUCOVID-001213	17-Aug-20	Marseille-1
OP646549	IHUCOVID-001215	17-Aug-20	Marseille-1
OP646550	IHUCOVID-001193	17-Aug-20	Marseille-3
OP646551	IHUCOVID-001229	17-Aug-20	Marseille-3
OP646552	IHUCOVID-001191	17-Aug-20	Marseille-4
OP646553	IHUCOVID-001189	17-Aug-20	Marseille-4
OP646554	IHUCOVID-001195	17-Aug-20	Marseille-5
OP646555	IHUCOVID-001206	17-Aug-20	Marseille-5
OP646556	IHUCOVID-001224	17-Aug-20	Marseille-7
OP646557	IHUCOVID-001283	18-Aug-20	Marseille-3
OP646558	IHUCOVID-001292	18-Aug-20	Marseille-5
OP646559	IHUCOVID-001279	18-Aug-20	Marseille-7
OP646560	IHUCOVID-001329	19-Aug-20	Marseille-5
OP646561	IHUCOVID-001341	19-Aug-20	Marseille-5
OP646562	IHUCOVID-001350	19-Aug-20	Marseille-7
OP646563	IHUCOVID-001336	19-Aug-20	Marseille-8
OP646564	IHUCOVID-001342	19-Aug-20	Marseille-2
OP646565	IHUCOVID-001345	19-Aug-20	Marseille-7
OP646566	IHUCOVID-001355	20-Aug-20	Marseille-4
OP646567	IHUCOVID-001897	26-Aug-20	Marseille-2
OP646568	IHUCOVID-001929	27-Aug-20	Marseille-6
OP646569	IHUCOVID-001976	27-Aug-20	Marseille-9
OP646570	IHUCOVID-001572	1-Sep-20	Marseille-5
OP646571	IHUCOVID-001592	1-Sep-20	Marseille-10
OP646572	IHUCOVID-001568	1-Sep-20	Marseille-10
OP646573	IHUCOVID-001625	10-Sep-20	Marseille-2
OP646574	IHUCOVID-001626	10-Sep-20	Marseille-5
OP646575	IHUCOVID-001627	10-Sep-20	Marseille-8
OP646576	IHUCOVID-001622	10-Sep-20	Marseille-10
OP646577	IHUCOVID-001628	11-Sep-20	Marseille-2
OP646578	IHUCOVID-001678	14-Sep-20	Marseille-2
OP646579	IHUCOVID-001672	14-Sep-20	Marseille-2
OP646580	IHUCOVID-001676	14-Sep-20	Marseille-10
OP646581	IHUCOVID-001665	15-Sep-20	Marseille-2
OP646582	IHUCOVID-001756	18-Sep-20	Marseille-2
OP646583	IHUCOVID-001758	19-Sep-20	Marseille-10
OP646584	IHUCOVID-002071	23-Sep-20	Marseille-2

Supplementary Table S2 (continued)

GenBank_ID	Sequence_ID	Collection date	Genotype
OP646585	IHUCOVID-002103	26-Sep-20	Marseille-8
OP646586	IHUCOVID-002627	9-Oct-20	Marseille-8
OP646587	IHUCOVID-002394	12-Oct-20	Marseille-9
OP646588	IHUCOVID-002401	13-Oct-20	Marseille-2
OP646589	IHUCOVID-002569	20-Oct-20	Marseille-8
OP646590	IHUCOVID-002653	20-Oct-20	Marseille-9
OP646591	IHUCOVID-002589	21-Oct-20	Marseille-9
OP646592	IHUCOVID-002725	9-Nov-20	Marseille-4
OP646593	IHUCOVID-002729	12-Nov-20	Marseille-4
OP646594	IHUCOVID-002732	17-Nov-20	Marseille-4
OP646595	IHUCOVID-003235	21-Jan-21	Alpha
OP646596	IHUCOVID-003219	22-Jan-21	Alpha
OP646597	IHUCOVID-003239	25-Jan-21	Alpha
OP646598	IHUCOVID-003247	28-Jan-21	Beta
OP646599	IHUCOVID-003178	2-Feb-21	Alpha
OP646600	IHUCOVID-003277	3-Feb-21	Alpha
OP646601	IHUCOVID-003288	10-Feb-21	Alpha
